# Intratumoral immunotherapy using a TLR2/3 agonist, L-pampo, induces robust antitumor immune responses and enhances immune checkpoint blockade

**DOI:** 10.1136/jitc-2022-004799

**Published:** 2022-06-28

**Authors:** Won Suk Lee, Dong Sung Kim, Jeong Hun Kim, Yoonki Heo, Hannah Yang, Eun-Jin Go, Jin Hyoung Kim, Seung Joon Lee, Byung Cheol Ahn, Jung Sun Yum, Hong Jae Chon, Chan Kim

**Affiliations:** 1 Laboratory of Translational Immuno-Oncology, Seongnam, Gyeonggi-do, Korea (the Republic of); 2 Medical Oncology, CHA Bundang Medical Center, CHA University School of Medicine, Seongnam, Gyeonggi-do, Korea (the Republic of); 3 Department of Biomedical Science, CHA University, Seongnam, Gyeonggi-do, Korea (the Republic of); 4 CHA Vaccine Institute, Seongnam, Gyeonggi-do, Korea (the Republic of)

**Keywords:** immunotherapy, tumor microenvironment, translational medical research

## Abstract

**Background:**

Toll-like receptors (TLRs) are critical innate immune sensors that elicit antitumor immune responses in cancer immunotherapy. Although a few TLR agonists have been approved for the treatment of patients with early-stage superficial cancers, their therapeutic efficacy is limited in patient with advanced invasive cancers. Here, we identified the therapeutic role of a TLR2/3 agonist, L-pampo (LP), which promotes antitumor immunity and enhances the immune checkpoint blockade.

**Methods:**

We generated LP by combining a TLR2 agonist, Pam3CSK4, with a TLR3 agonist, Poly (I:C). Immune responses to stimulation with various TLR agonists were compared. Tumor-bearing mice were intratumorally treated with LP, and their tumor sizes were measured. The antitumor effects of LP treatment were determined using flow cytometry, multiplexed imaging, and NanoString nCounter immune profiling. The immunotherapeutic potential of LP in combination with α-programmed cell death protein-1 (PD-1) or α-cytotoxic T-lymphocytes-associated protein 4 (CTLA-4) was evaluated in syngeneic MC38 colon cancer and B16F10 melanoma.

**Results:**

The LP treatment induced a potent activation of T helper 1 (Th1) and 2 (Th2)-mediated immunity, tumor cell apoptosis, and immunogenic tumor cell death. Intratumoral LP treatment effectively inhibited tumor progression by activating tumor-specific T cell immunity. LP-induced immune responses were mediated by CD8^+^ T cells and interferon-γ, but not by CD4^+^ T cells and CD25^+^ T cells. LP simultaneously activated TLR2 and TLR3 signaling, thereby extensively changing the immune-related gene signatures within the tumor microenvironment (TME). Moreover, intratumoral LP treatment led to systemic abscopal antitumor effects in non-injected distant tumors. Notably, LP treatment combined with ɑPD-1 and ɑCTLA-4 further enhanced the efficacy of monotherapy, resulting in complete tumor regression and prolonged overall survival. Furthermore, LP-based combination immunotherapy elicited durable antitumor immunity with tumor-specific immune memory in colon cancer and melanoma.

**Conclusions:**

Our study demonstrated that intratumoral LP treatment improves the innate and adaptive antitumor immunity within the TME and enhances the efficacy of αPD-1 and αCTLA-4 immune checkpoint blockade.

**WHAT IS ALREADY KNOWN ON THIS TOPIC**

- The toll-like receptor (TLR) signaling pathway is an important link between the innate and adaptive immunity, and induces anticancer immune responses. L-pampo (LP), a TLR2/3 agonist, revealed to be an efficient vaccine adjuvant, but its antitumor efficacy remains to be elucidated.

**WHAT THIS STUDY ADDS**

- Intratumoral administration of LP promotes the remodeling of the tumor microenvironment by activating tumor-specific T-cell immunity. Notably, the combination immunotherapy with LP, ɑ-programmed cell death protein-1 (PD-1), and ɑ-cytotoxic T-lymphocytes-associated protein 4 (CTLA-4) elicits the most potent antitumor response, leading to complete tumor regression and long-term overall survival.

**HOW THIS STUDY MIGHT AFFECT RESEARCH, PRACTICE, OR POLICY**

- Intratumoral LP treatment may be a promising therapeutic strategy to enhance innate and adaptive antitumor immunity within the tumor and potentiate the immunotherapeutic efficacy of immune checkpoint inhibitors.

## Background

Advances in cancer immunology have revealed the potential of various immunotherapeutics involving immune checkpoint inhibitors (ICIs).^1–4^ However, the use of these strategies still has limitations, including primary and acquired resistance, and immune-related adverse events, despite their potent and durable antitumor efficacy.^5–9^ Therefore, the optimization of cancer immunotherapy remains necessary.^10 11^

The toll-like receptor (TLR) signaling pathway represents a critical interface between innate and adaptive immunity, thereby enhancing anticancer immune responses.^12 13^ TLRs are pattern recognition receptors (PRRs) that are membrane-bound at the cell surface or endosomes, and are stimulated by various pathogen-associated molecular patterns (PAMPs) and damage-associated molecular patterns (DAMPs).^14^ Extracellular TLRs (TLR1, TLR2, TLR4, TLR5, TLR6, and TLR10) recognize microbial molecules, while intracellular TLRs (TLR3, TLR7, TLR8, and TLR9) recognize single-stranded or double-stranded RNA and CpG DNA.^15 16^ Stimulation of TLR signaling activates antigen-presenting cells (APCs), thereby inducing cytokine secretion and co-stimulatory signals and promoting the antigen presenting capacity.^17 18^ TLR agonists have been evaluated in preclinical studies and show potent antitumor efficacies by enhancing both innate and adaptive immunity.^19^ However, the clinical development of TLR agonists is impeded by the induction of systemic immune-related toxicities such as cytokine release syndrome, and limited efficacy in patients with advanced cancers.^20^ Therefore, only two TLR agonists have been approved for treating patients with early-stage superficial cancers: intravesical BCG (TLR2/4 agonist) for superficial bladder cancer and topical imiquimod (TLR7 agonist) for basal cell carcinoma.^21 22^ Therefore, it is necessary to develop a potent TLR-based therapy that is effective even in patients with advanced cancers.

L-pampo is a TLR2 and TLR3 agonist composed of Pam3CSK4 and polyinosinic:polycytidylic acid, and can improve the efficacy of vaccines when used as an adjuvant.^23^ It can simultaneously activate TLR3, thus inducing type I interferon (IFN) and polarizing T helper 1 (Th1) immunity.^24^ L-pampo also simultaneously elicits T helper 2 (Th2) immune responses via the TLR2 signaling pathway.^24^ A previous study revealed that L-pampo can enhance the efficacy of vaccines as an immune adjuvant by promoting antibody production and enhancing functional CD4^+^ T cell responses in an ovalbumin or hepatitis B virus (HBV) immunization mouse model.^25^ L-pampo is well-tolerated without inducing considerable toxicity when used as an immune adjuvant in combination with HBV antigen in a phase I clinical trial (NCT02693652). However, its antitumor efficacy as an independent antitumor drug, and not as an immune adjuvant, has not been elucidated yet. Moreover, the combination immunotherapy of L-pampo with ICIs has not been investigated.

Here, we investigated the effects of intratumoral L-pampo (hereafter referred to as LP) treatment on immunogenic tumor cell death and tumor immune microenvironment to study its role in potentiating the efficacy of various ICIs.

## Methods

### Mice and cell lines

Male C57BL/6 mice were purchased from Orient Bio (Korea). The mice were housed in a specific pathogen-free facility at CHA Bio Complex (Korea). The Raw264.7 macrophage, HEK293 kidney cell, and HeLa cervix cancer cell lines were purchased from American Type Culture Collection (ATCC). The MC38 colon cancer cell line and B16F10 melanoma cell line were obtained from the National Cancer Center (Korea). These cells were maintained in DMEM or MEM, supplemented with 10% fetal bovine serum (FBS) and 1% penicillin/streptomycin, and incubated at 37°C and 5% CO_2_.

### TLR agonists

The following TLR agonists were used: Alum (Thermo Fisher Scientific); monophosphoryl lipid A (MPLA) (Invivogen); CpG oligodeoxynucleotide (ODN) 2395, type C specific for human and murine (Invivogen); Pam3 (Creative Biolabs); Poly (I:C) (Yamasa Corporation); and LP (CHA Vaccine Institute). The concentrations for cytokine production used here were Alum at 100 µg/mL, MPLA at 5 µg/mL, CpG ODN 2395 at 100 µg/mL, Pam3 at 100 µg/mL, Poly (I:C) at 100 µg/mL, and LP at 45 µg/mL. Furthermore, LP (45 µg/mL) and the equivalent amounts of Pam3 and Poly (I:C) in LP were used to measure apoptosis and DAMPs.

### Tumor models and treatment regimens

Tumors were implanted via subcutaneous injection of 1×10^5^ MC38 or 5×10^5^ B16F10 cells into the right flank of mice. When the tumor volume exceeded 50 mm^3^, mice were intratumorally injected four times with 50 µL of LP or phosphate-buffered saline (PBS) every 3 days. For the cell depletion experiment, the mice received intraperitoneal administrations of 8 mg/kg of the following neutralizing antibodies at given time points: anti-CD8 (clone 53–6.72; BioXCell, New Hampshire, USA), anti-CD4 (clone GK1.5; BioXCell), anti-CD25 (clone PC-61.5.3; BioXCell), or anti-IFN-γ (clone XMG1.2; BioXCell). For immune checkpoint blockade, mice were treated with intraperitoneal injections of anti-programmed cell death protein-1 (PD-1) (8 mg/kg, clone J43; BioXCell) and/or anti-cytotoxic T-lymphocytes-associated protein 4 (CTLA-4) (4 mg/kg, clone 9D9; BioXCell) antibody at the given time points. To evaluate the abscopal effects, 1×10^5^ MC38 cells were subcutaneously implanted into the right flank, and the same amount was subcutaneously implanted into the left flank 4 days later. Mice with complete tumor regression were rechallenged with 1×10^5^ MC38 or 5×10^5^ B16F10 tumor cells in the left flank, and tumor growth was monitored. The tumors were measured using a digital caliper, and tumor volumes were calculated using the following formula: 1/2 × (length×width^2^). For survival analysis, the mice were euthanized when the tumor volume exceeded 2000 mm^3^ or when the mice became moribund.

### RNA isolation and NanoString gene expression analysis

For NanoString gene expression analysis, total RNA was extracted from tumor tissues using TRIzol reagent (Invitrogen, Massachusetts, USA) and purified using ethanol. The quality and concentration of RNA were examined using a fragment analyzer (Advanced Analytical Technologies, Iowa, USA). Immune profiling was performed using a digital multiplexed NanoString nCounter PanCancer Immune Profiling mouse panel (NanoString Technologies, Washington, USA) with 100 ng of total RNA isolated from tumor samples, as described previously.^26 27^

### Flow cytometry analysis

For flow cytometry analysis, tumor tissues were minced and incubated for 1 hour at 37°C in a digestion buffer comprizing 40 µg/mL DNase I (Roche) and 2 mg/mL collagenase D (Roche, Switzerland). Cell suspensions were filtered through a 70 µm cell strainer (Corning, New York, USA) and incubated for 3 min at room temperature in ACK lysis buffer (Gibco, Massachusetts, USA). After washing with fluorescence-activated cell sorting (FACS) buffer (1% FBS in PBS), the cells were further filtered through a nylon mesh. Next, the cells were incubated on ice for 30 min in Fixable Viability Dye eFluor 450 (Invitrogen) to exclude dead cells. The cells were washed with FACS buffer and incubated on ice for 30 min in FACS buffer containing primary antibodies targeting CD45 (30-F11, Invitrogen), CD3 (17A2 or 145–2 C11, Invitrogen), CD8a (53–6.7 or KT15, Invitrogen), CD4 (RM4-5, Invitrogen), CD25 (PC61.5, Invitrogen), ICOS (7E.17G9, Invitrogen), CD11b (M1/70, Invitrogen), Ly-6G (1A8-Ly6g, Invitrogen), Ly-6C (HK1.4, Invitrogen), F4/80 (BM8, Invitrogen), CD62L (MEL-14, Invitrogen), or CD44 (IM7, Invitrogen). Cells were further permeabilized using a Foxp3 Staining Buffer kit (Invitrogen) and stained for Foxp3 (FJK-16s, Invitrogen), granzyme B (NGZB, Invitrogen), iNOS (CXNFT, Invitrogen), or arginase 1 (A1exF5, Invitrogen). To identify the tumor-specific CD8 T cells, the cells from tumors, spleens, and lymph nodes were stained with H-2K^b^ MuLV p15E Tetramer-KSPWFTTL (MBL International, Massachusetts, USA). The stained cells were analyzed using a CytoFLEX flow cytometer (Beckman Coulter, California, USA), and the data were analyzed with the FlowJo software (Tree Star, Oregon, USA).

### Co-culture system

For co-culture experiment, splenocytes were isolated from C57BL/6 mice and purified using Mouse T cell Isolation Kit (Thermo Fisher Scientific). After the purification, T cells were co-cultured with Raw264.7 macrophages at a ratio of 3:1 in a 24-well plate and treated with LP. After the incubation for 72 hours, the culture supernatant were collected for cytokine measurements.

### Histological analyses

The tumor samples were fixed in 1% paraformaldehyde, dehydrated overnight in 20% sucrose solution, and frozen in OCT compound (Leica, Germany). The frozen samples were sectioned into 50 μm-thick slices, which were permeabilized in 0.3% PBS-T (Triton X-100 in PBS) and blocked with 5% normal goat serum in 0.1% PBS-T for 30 min at room temperature. Subsequently, the samples were incubated overnight with the following primary antibodies: anti-high-mobility group box 1 (HMGB1) (rabbit, Abcam), anti-CRT (rabbit, Abcam), anti-CD11c (hamster, BD pharmingen), anti-iNOS (rabbit, Abcam), anti-CD206 (rat, Invitrogen), anti-CD8 (rat, clone 53–6.7; BD Biosciences, New Jersey, USA), anti-CD31 (hamster, clone 2H8, Millipore, Massachusetts, USA; rabbit, Abcam, UK), anti-Caspase-3 (rabbit, R&D Systems, Minnesota, USA), or anti-programmed death ligand-1 (PD-L1) (rabbit, clone 28–8, Abcam). After several washes, the samples were incubated for 2 hours at room temperature with the following secondary antibodies: fluorescein isothiocyanate (FITC)- or Cy3-conjugated anti-rabbit IgG (Jackson ImmunoResearch, Pennsylvania, USA), FITC- or Cy3-conjugated anti-rat IgG (Jackson ImmunoResearch), or Cy3-conjugated anti-hamster IgG (Jackson ImmunoResearch). Cell nuclei were counterstained with 4′,6-diamidino-2-phenylindole (Invitrogen). Finally, the samples were mounted using fluorescent mounting medium (DAKO, Denmark), and images were acquired using a Zeiss LSM 880 microscope (Carl Zeiss, Germany).

### Morphometric analyses

Density measurements of HMGB1^+^ cell area, CRT^+^ cell area, DCs, M1-like macrophages, M2-like macrophages, blood vessels, T lymphocytes, apoptotic cells, and PD-L1^+^ cell area were performed using ImageJ software (http://rsb.info.nih.gov/ij), as previously described.^28 29^

### IFN-γ enzyme-linked immunospot assay

To determine the activation of tumor-specific T cells, splenocytes were isolated 3 days after the last treatment. Splenocytes were then incubated with MC38 tumor cells at a ratio of 1:10 in the anti-mouse IFN-γ-precoated plate for 24 hours (37°C with 5% CO_2_). After washing, the plates were stained with 1 µg/mL biotinylated anti-mouse IFN-γ antibody (R4-6A2-biotin) for 2 hours at room temperature, followed by incubation with streptavidin-ALP for 1 hour at room temperature. Finally, after the addition of the BCIP/NBT-plus substrate solution, spot density was measured using the ImageJ software (http://imagej.net/Fiji).^30^

### Cell apoptosis assay

To measure apoptosis, 1.5×10^5^ MC38 and HeLa cells were plated into each well of a six-well plate and treated with TLR agonists for 24 hours at 37°C. Cell apoptosis and caspase-9 activity were determined using the Annexin V Apoptosis Detection Kit (BioLegend) and colorimetric assay kit (Abcam), respectively. The samples were analyzed using a CytoFLEX flow cytometer (Beckman Coulter) and a microplate reader (Thermo Fisher Scientific).

### ELISA

To quantify cytokines and DAMPs, 1×10^5^ cells (Raw264.7, HEK293, MC38, and HeLa) were seeded into a 12-well plate overnight and treated with TLR agonists or 20 µM oxaliplatin, a potent immunogenic cell death inducer, in complete culture medium. Culture supernatants were harvested, and the levels of the tumor necrosis factor (TNF)-α (BD Biosciences), interleukin (IL)-6 (BD Biosciences), HMGB1 (IBL International), and calreticulin (MyBioSource) were quantified using an ELISA kit, following the manufacturer’s instructions.

### Statistical analysis

Statistical analyses were performed using GraphPad Prism V.7.0 software (GraphPad Software, California, USA) and PASW statistics V.18 (SPSS; IBM, New York, USA). Values are presented as the mean±SD unless otherwise indicated. The Shapiro-Wilk normality test was performed for all data sets to analyze whether each data set followed a normal distribution pattern. Parametric tests, such as Student’s t-test and one-way analysis of variance, were performed to analyze data following a normal distribution. Non-parametric tests, such as the Mann-Whitney U test and Kruskal-Wallis test, were performed to analyze data showing abnormal distribution owing to small sample size. Survival curves were plotted using the Kaplan-Meier method, and statistical differences between the curves were determined using the log-rank test. The level of significance was set at p<0.05.

## Results

### LP treatment can induce the potent activation of Th1 and Th2-mediated immunity, tumor cell apoptosis, and immunogenic tumor cell death

**Figure 1:**
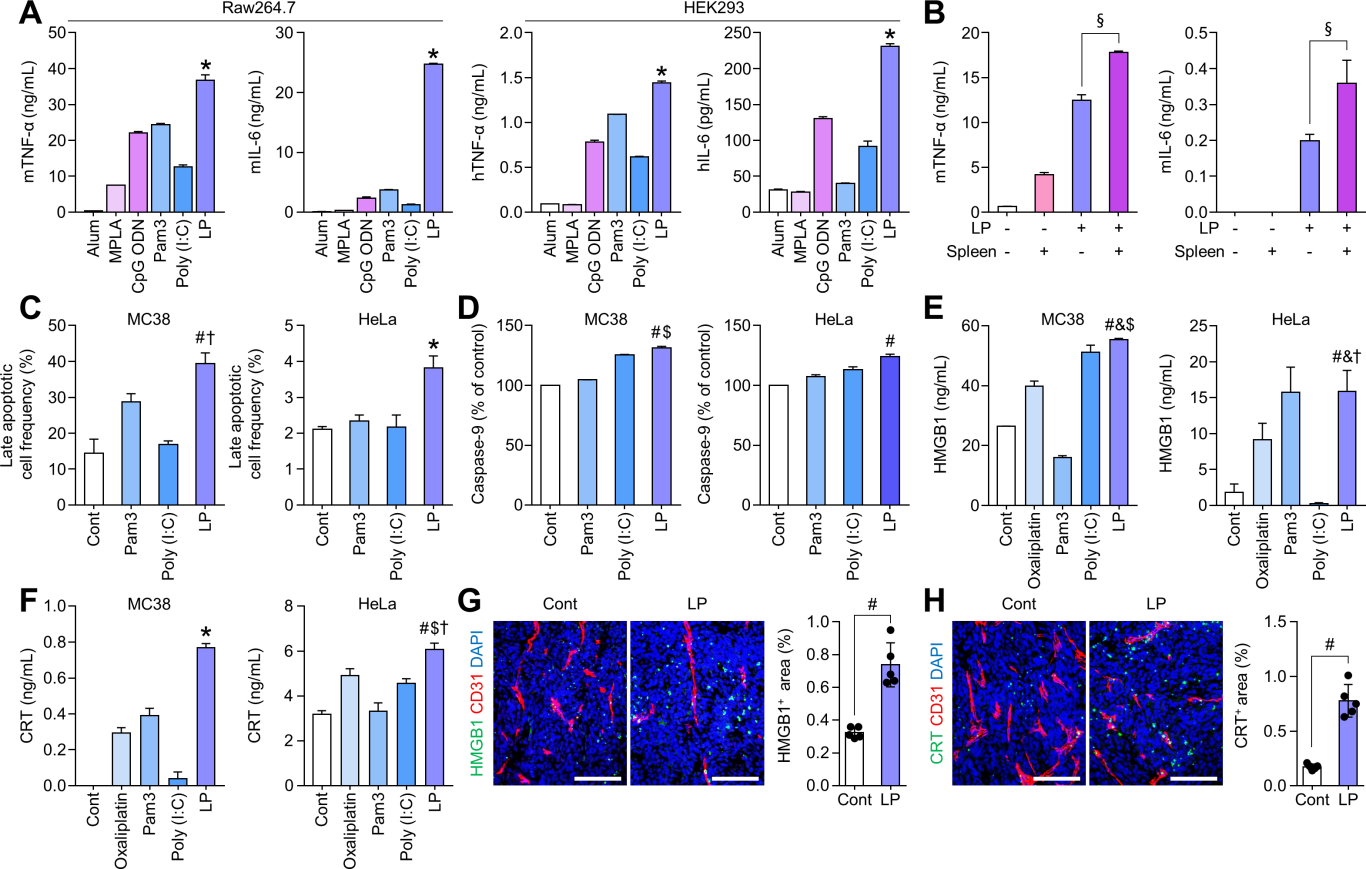
LP enhances the TLR-mediated activation of cellular immune responses. (A) Comparison of the levels of TNF-α and IL-6 in murine Raw264.7 and human HEK293 cells. (B) Comparison of the levels of TNF-α and IL-6 in co-culture of murine Raw264.7 and murine splenocytes. (C) Comparison of Annexin V^+^PI^+^ apoptotic cells in murine MC38 and human HeLa cells. (D) Comparison of activated caspase-9 levels in murine MC38 and human HeLa cells. (E and F) Comparison of HMGB1 and CRT secretion in MC38 and HeLa cells. (G and H) Representative confocal images and comparison of HMGB1^+^ and CRT^+^ immunogenic cell death within the tumor microenvironment after intratumoral treatment of phosphate-buffered saline or LP (50 µg). Pooled data from two independent experiments with n=5 per group (G and H). Values are expressed as the mean±SD. *P<0.05 versus all other groups; ^§^p<0.05 versus macrophage alone; ^#^p<0.05 versus control; ^$^p<0.05 versus Pam3, ^†^p<0.05 versus Poly (I:C); ^&^p<0.05 versus oxaliplatin. Student’s t-test was used (A–H). Scale bars, 100 µm. Alum, aluminum hydroxide; CpG ODN, CpG oligodeoxynucleotide; CRT, calreticulin; HMGB1, high-mobility group box 1; IL-6, interleukin-6; LP, L-pampo; MPLA, monophosphoryl lipid A; Pam3, Pam3CSK4; Poly (I:C), polyinosinic-polycytidylic acid; TLR, toll-like receptors; TNF, tumor necrosis factor.

We determined the levels of TNF-α (Th1 cytokine) and IL-6 (Th2 cytokine) in murine antigen-presenting Raw264.7 and human HEK293 cells on stimulation with various TLR agonists: MPLA (TLR4), CpG ODN (TLR9), Pam3 (TLR2), Poly (I:C) (TLR3), and LP (TLR2 and TLR3). LP showed the most upregulation of TNF-α and IL-6 compared with other TLR agonists (figure 1A). Moreover, both cytokines were significantly increased when macrophages are co-cultured with lymphocytes compared with macrophage alone. Therefore, macrophage and lymphocytes co-operated to elicit Th1 and Th2 response on stimulation with LP (figure 1B). In addition, LP induced apoptosis in both murine MC38 colon cancer and human HeLa cervical cancer cells (figure 1C, D). Moreover, the levels of the immunogenic cell death markers, HMGB1 and calreticulin, were also remarkably upregulated in LP-treated apoptotic tumor cells compared with some other TLR agonists in vitro (figure 1E, F). Consistently, intratumoral LP treatment strongly induced immunogenic cell death in vivo in tumor tissues (figure 1G, H). Collectively, LP treatment induced the simultaneous activation of Th1 and Th2-mediated immunity, tumor cell apoptosis, and immunogenic tumor cell death more strongly than most of the other TLR agonists.

### Intratumoral LP treatment suppressed tumor growth by activating tumor-specific T cell immunity

**Figure 2:**
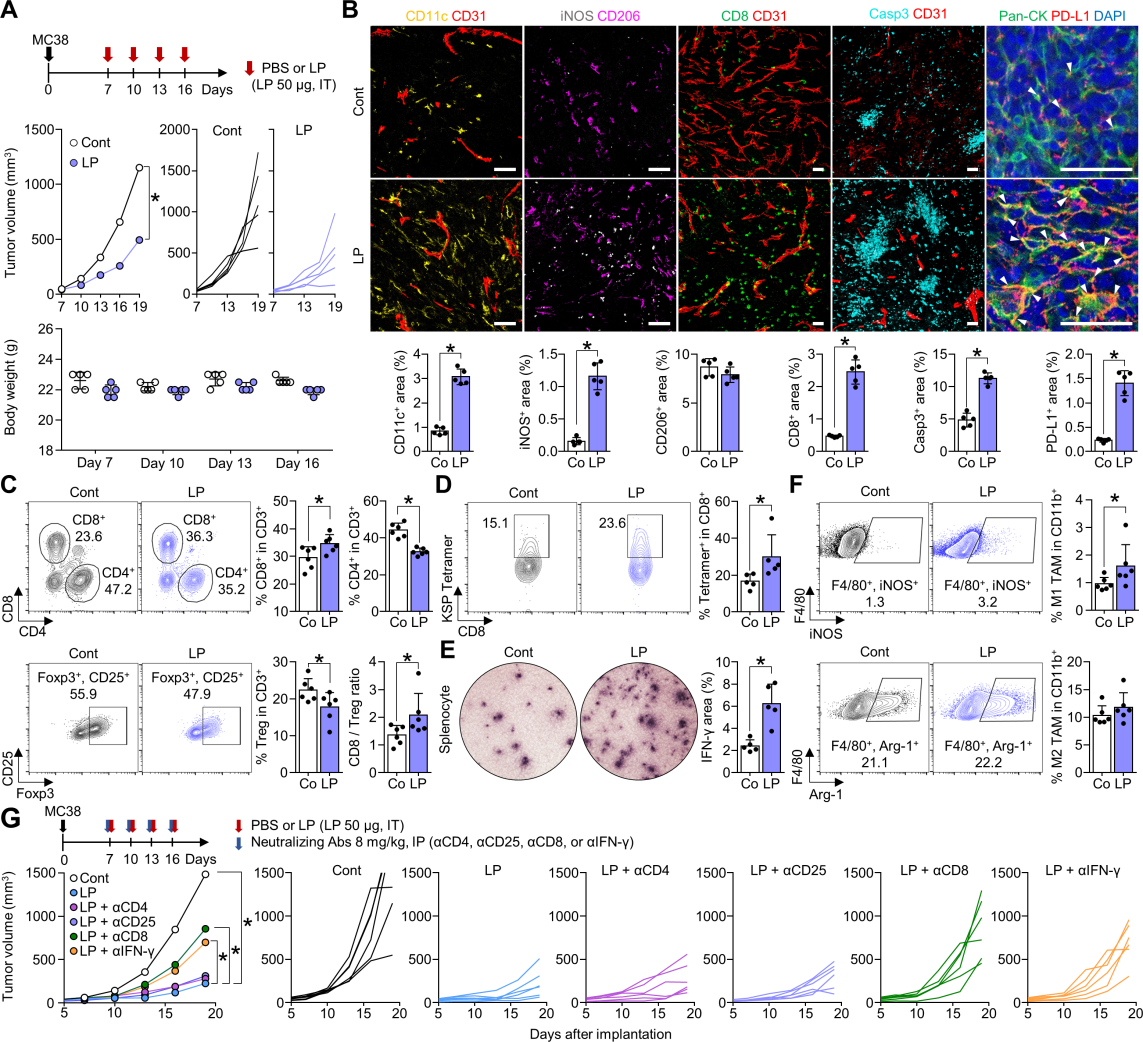
Intratumoral LP treatment promotes CD8^+^ T-cell activation that inhibits tumor growth. MC38 tumor cells were subcutaneously implanted into mice and treated with intratumoral injection of PBS or LP (A–F) and were intraperitoneally injected with various neutralizing antibodies against CD4 (αCD4), CD25 (αCD25), CD8 (αCD8), or IFN-γ (αIFN-γ) (G). (A) Comparison of tumor growth and body weight in mice treated with PBS or LP. Arrows indicate treatments. (B) Representative images and comparisons of CD11c^+^ DCs, iNOS^+^ M1-like macrophages, CD206^+^ M2-like macrophages, CD8^+^ T-cells, CD31^+^ tumor vasculatures, caspase-3^+^ apoptotic cells, and PD-L1^+^ cells within tumors. Arrowheads indicate PD-L1 expression in pan-cytokeratin (Pan-CK)^+^ tumor cells. (C) Representative plots and comparisons of CD8^+^ T cells, CD4^+^ T cells, and CD4^+^Foxp3^+^CD25^+^ (Treg) in tumors. (D) Representative plot showing the tumor-specific KSP^+^ fraction in CD8^+^ T cells. (E) Images and comparisons of IFN-γ ELISPOT in splenocytes from PBS-treated and LP-treated mice. (F) Representative plots and comparisons of M1-like (F4/80^+^iNOS^+^) and M2-like (F4/80^+^Arg-1^+^) tumor-associated macrophages in tumors. (G) Treatment schedule for CD4, CD25, CD8, and IFN-γ depletion. Comparison of mean and individual tumor growth curves over time. Pooled data from two independent experiments with n=5 per group (A, B, D and E) and n=6 per group (C, F and G). Values are expressed as the mean±SD. *P<0.05, versus control. A two-tailed Student’s t-test was used (B–G). Scale bars, 50 µm. Arg-1, arginase-1; CD8, cluster of differentiation 8; DC, dendritic cell; ELISPOT, enzyme-linked immunospot; IFN, interferon; LP, L-pampo; PBS, phosphate-buffered saline; PD-L1, programmed death-ligand 1.

To determine the antitumor efficacy of LP, MC38 tumor-bearing mice were intratumorally injected with PBS or LP. After four consecutive injections, LP treatment inhibited MC38 tumor growth by 57.2% compared with that observed in PBS-treated mice. There were no significant differences in body weight among PBS-treated mice and LP-treated mice (figure 2A). Additionally, histological analyses revealed that LP treatment increased the proportions of CD11c^+^ dendritic cells (DCs) by 3.7-fold, iNOS^+^ M1-like macrophages by 7.3-fold, CD8^+^ T cells by 5.2-fold, Casp3^+^ apoptotic cells by 2.3-fold, and PD-L1^+^ tumor cells by 6.1-fold within tumors compared with those in the control group (figure 2B). Flow cytometric analyses also revealed that the proportion of CD8^+^ T cells increased by 1.2-fold in the LP group compared with that in the control group, while the proportion of CD4^+^ T cells decreased by 26.4%. Notably, the proportion of CD4^+^CD25^+^Foxp3^+^ regulatory T cells were reduced by 20.6% and the CD8/Tregs ratio increased by 1.5-fold after LP treatment (figure 2C).

Next, we quantified MC38 tumor-specific cytotoxic T cells using MC38 tumor dominant epitope (KSPWFTTL-H-2Kb (KSP) peptide) tetramer, and found that the proportion of MC38 tumor-specific CD8^+^ T cells increased by 1.8-fold within LP-treated (figure 2D). In addition, IFN-γ enzyme-linked immunospot assays showed a 2.6-fold increase in IFN-γ secretion from LP-treated T cells compared with that in control T cells (figure 2E). The level of the M1 marker iNOS increased by 1.7-fold after LP treatment, while M2 marker Arg-1 showed no significant changes (figure 2F). Therefore, LP induced potent antitumor efficacy by activating tumor-specific CD8^+^ T cells and enhancing M1-like macrophages within the tumor microenvironment (TME).

To identify the components of the immune system responsible for the efficacy of LP, we investigated the impact of various neutralizing antibodies (anti-CD4, anti-CD25, anti-CD8, and anti-IFN-γ) on the efficacy of LP. Although depletion of CD4 or CD25 did not affect the antitumor efficacy of LP, depletion of CD8 or IFN-γ significantly reduced the efficacy of LP treatment. Therefore, CD8^+^ T cells and IFN-γ play important roles in LP-mediated immune responses within the tumor (figure 2G).

### LP reprogrammed the tumor immune landscape via dual targeting of TLR2 and TLR3 signaling

**Figure 3:**
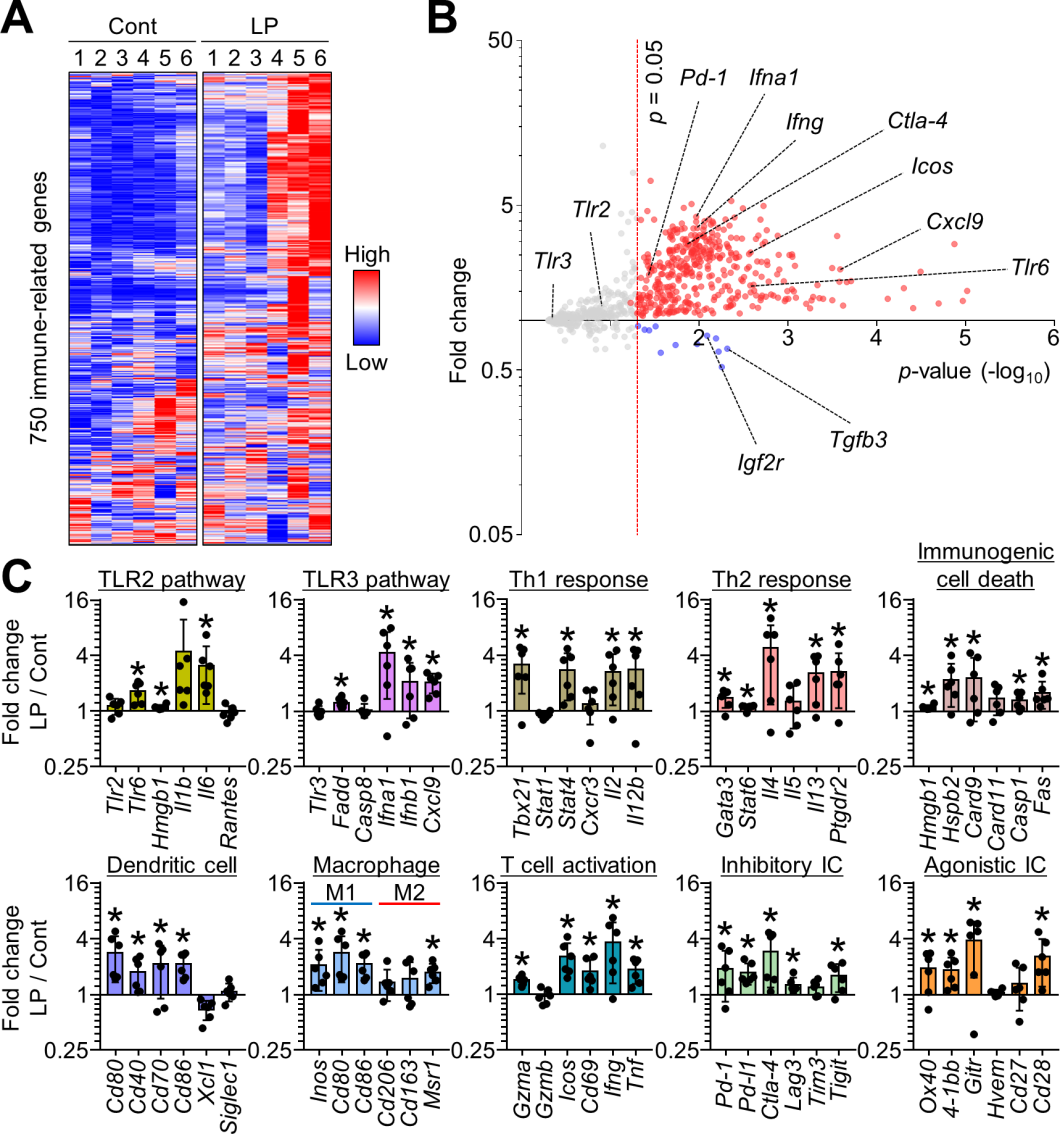
LP induces extensive immune remodeling of the TME via blocking of TLR2 and TLR3 signaling pathways. MC38-tumor bearing mice were intratumorally injected with PBS or LP. (A) Heatmap of NanoString immune-related genes. Red color represent upregulated and blue color represent downregulated genes, respectively. (B) Volcano plot showing changes in gene expression profiles in LP-treated tumors. Red line indicates p<0.05. (C) Comparisons of gene expression related to TLR2 pathway, TLR3 pathway, T helper 1 response, T helper 2 response, immunogenic cell death, dendritic cells, macrophages, T cell activation, inhibitory ICs, and agonistic ICs. Pooled data from two independent experiments with n=6 per group (A–C). Values are expressed as the mean±SD. *P<0.05, versus control. Two-tailed Student’s t-tests were performed (C). ICIs, immune checkpoint inhibitors; LP, L-pampo; PBS, phosphate-buffered saline; TLR, toll-like receptor; TME, tumor microenvironment.

To comprehensively elucidate LP-induced immune remodeling within the tumor, immune-related gene signatures were analyzed using NanoString PanCancer Immune Profiling of MC38 tumor tissues. LP treatment induced widespread upregulation of immune-related genes (figure 3A, B). Notably, TLR2 and TLR3 responsive genes were upregulated after LP treatment. In addition, genes related to Th1 and Th2 responses, immunogenic cell death, DCs, M1 macrophage polarization, and T-cell activation were significantly upregulated after LP treatment. Various inhibitory immune checkpoints (*Pd-1*, *Pd-l1*, *Ctla-4*, *Lag-3*, and *Tigit*) and agonistic immune checkpoints (*Ox40*, *4-1bb*, *Gitr*, and *Cd28*) were also upregulated in LP-treated tumors compared with those in control tumors (figure 3C).

### Local LP injection elicited systemic antitumor immune responses in distant tumors

**Figure 4:**
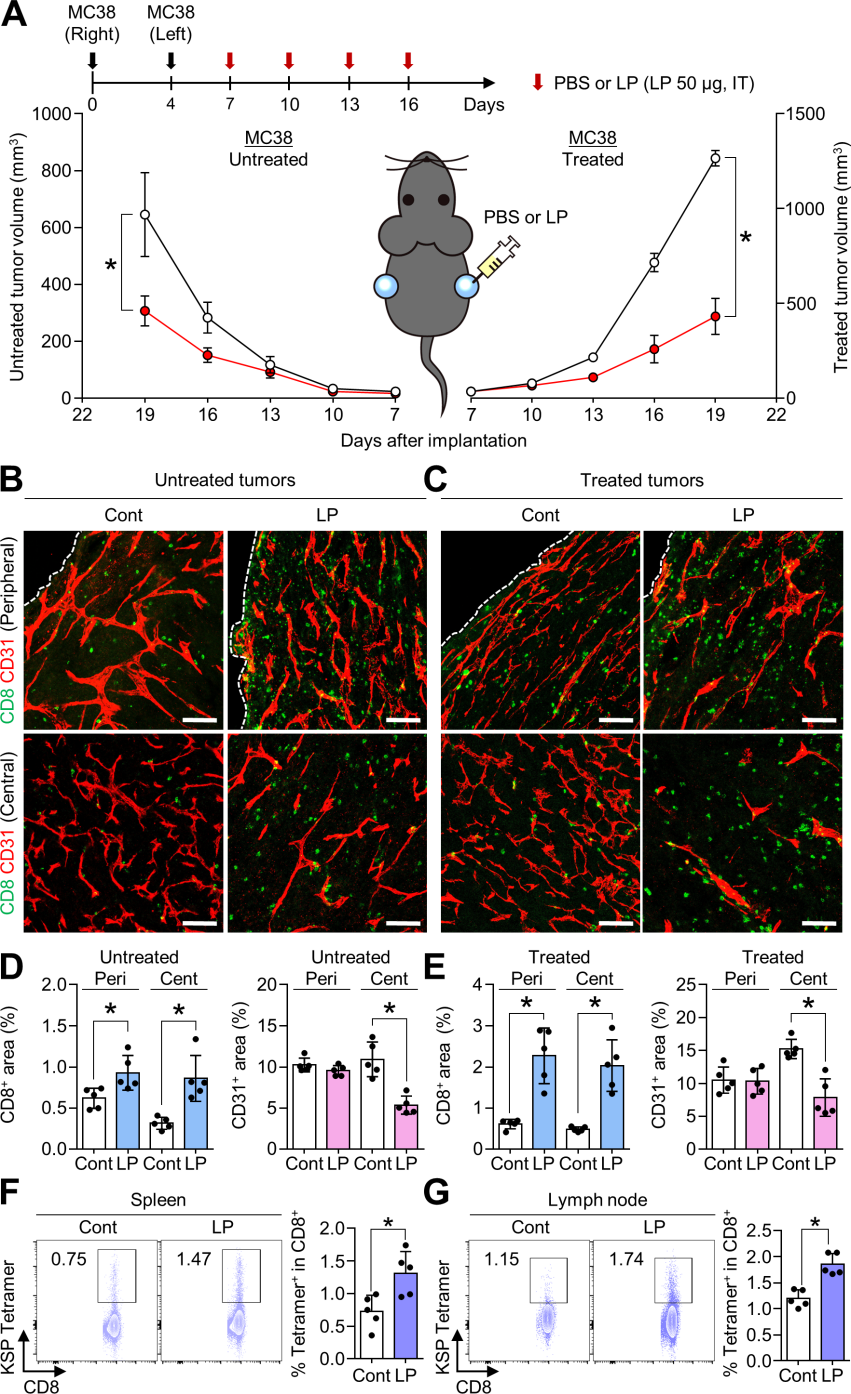
Local treatment of LP elicits systemic immune responses. Mice were subcutaneously implanted with MC38 tumor cells in both the right flank and left flank, and intratumorally injected with PBS or LP. (A) Growth curves of LP-injected MC38 tumors and non-injected MC38 tumors. (B and C) Representative images of CD8^+^ T cells and CD31^+^ tumor vasculatures in LP-injected and non-injected tumors. (D and E) Comparison of CD8^+^ T cells and CD31^+^ tumor vasculatures in LP-injected and non-injected tumors. (F and G) Representative plot showing the tumor-specific KSP^+^ fraction of CD8^+^ T cells in spleens and lymph nodes. Pooled data from three independent experiments with n=8 per group (A) and n=5 per group (D–G). Values are expressed as the mean±SEM. *P<0.05, versus control. Two-tailed Student’s t-test was used (D–G). Scale bars, 100 µm. CD8, cluster of differentiation 8; LP, L-pampo; PBS, phosphate-buffered saline.

Since most patients with advanced cancers have multiple systemic metastases, the control of a single tumor mass has limited therapeutic implications in clinical practice. Therefore, we investigated whether the local immune response induced by intratumoral LP treatment could be expanded to systemic immune responses that can control distant tumors that are not directly injected with LP. To generate the bilateral tumor model, MC38 tumor cells were subcutaneously implanted into the right flank of mice, and 4 days later, MC38 tumor cells were implanted into the left flank. LP or PBS was then intratumorally administered to the right flank tumor alone. After four consecutive injections, LP treatment suppressed the tumor in the right tumor by 65.9%, and more importantly, it suppressed the growth of the tumor in the left flank by 52.5% (figure 4A). In addition, the proportion of intratumoral CD8^+^ T cells increased in the peritumoral and central areas of both LP-injected and uninjected tumors compared with that in control tumors. Moreover, CD31^+^ tumor vascular densities were reduced in the central areas of both tumors in the right and left flanks of LP-treated mice (figure 4B–E). Next, we performed the KSP (KSPWFTTL) tetramer assay to evaluate the MC38 tumor-specific T cells in spleens and lymph nodes. The results showed that CD8^+^ T cells specific to MC38-dominant KSP peptide are increased in the lymphoid organs after LP treatment (figure 4F, G). Overall, local LP treatment could effectively elicit systemic antitumor T cell immune responses in distant tumors.

### Combination immunotherapy of LP with ICIs further enhanced antitumor efficacy and induced complete tumor regression

**Figure 5:**
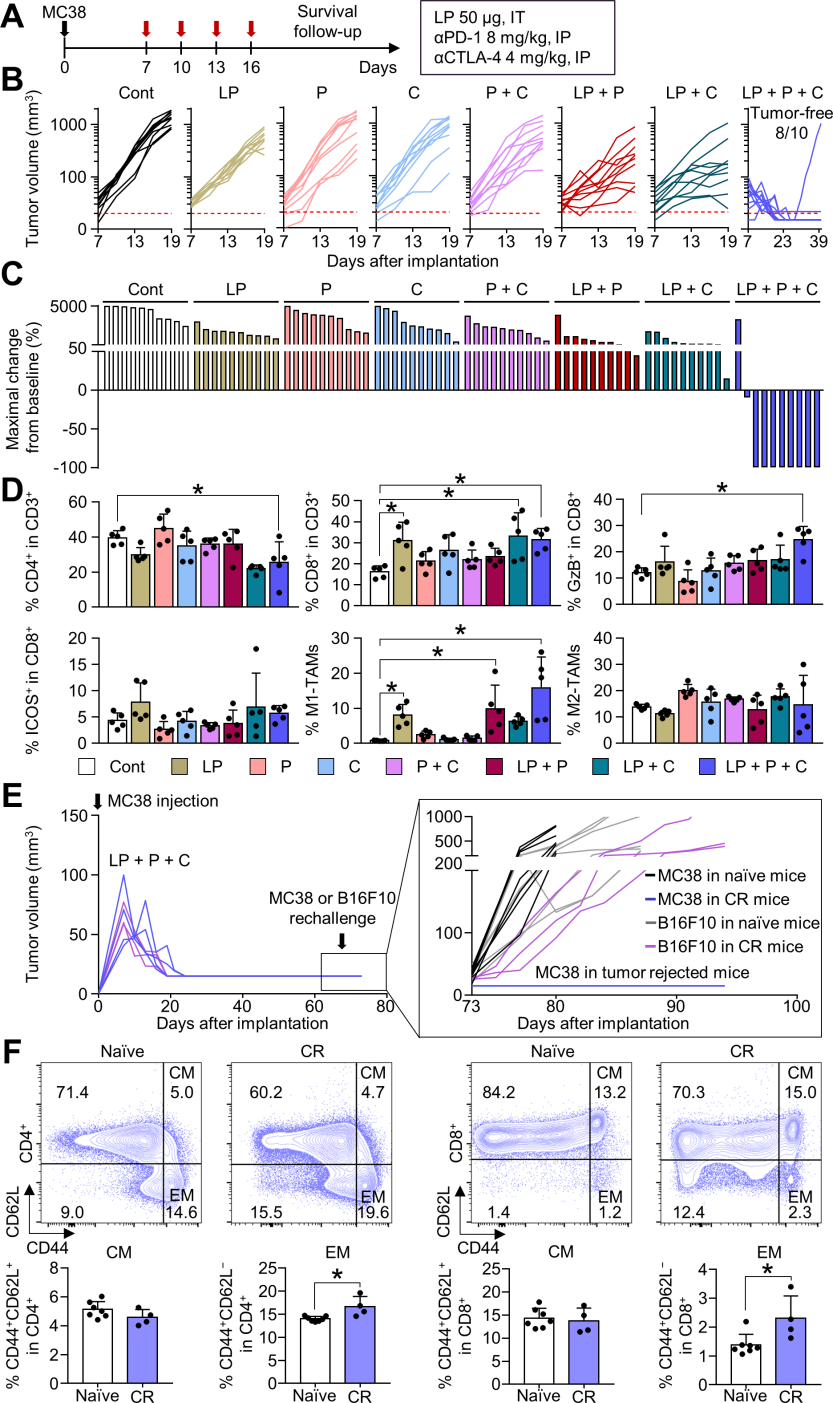
Combination treatment of LP with immune checkpoint inhibitors (ICIs) (αPD-1 and αCTLA-4) induces tumor regression. MC38-tumor bearing mice were treated with LP, αPD-1 (P), and/or αCTLA-4 (C). (A) Schematic diagram of the treatment schedule. (B) Comparison of MC38 tumor growth. The number of tumor-free mice is indicated. (C) Waterfall plots showing the percentage changes in each tumor volume at the end of the experiment compared with the baseline. (D) Comparisons of CD4^+^ T cells, CD8^+^ T cells, CD8^+^GzB^+^ cells, CD8^+^ICOS^+^ cells, iNOS^+^ M1-like TAM, and Arg-1^+^ M2-like TAM fractions in tumors. (E) Comparison of tumor growth after the injection of MC38 colon cancer or B16F10 melanoma cells into naïve mice or mice with complete tumor regression. (F) Representative plot and comparison of the central memory (CM, CD44^+^CD62L^+^) and effector memory (EM, CD44^+^CD62L^−^) T cells among CD4^+^ and CD8^+^ T cells within the spleen. Pooled data from three independent experiments with n=10 per group (B and C), n=5 per group (D), and n=3 to 7 per group (E and F). Values are presented as mean±SD *p<0.05 versus control. Two-tailed Student’s t-test was used (D and F). Arg-1, arginase-1; CR, complete tumor regression; CTLA-4, cytotoxic T-lymphocytes-associated protein 4; LP, L-pampo; PD-1, programmed cell death protein-1; TAM, tumor-associated macrophage.

Although intratumoral LP treatment elicited a potent antitumor T-cell immunity, the repeated LP treatment could also elicit a counteractive upregulation of immune checkpoints, such as PD-1 and CTLA-4, as a negative feedback mechanism (figure 3C). Since these immune checkpoints may trigger adaptive resistance to LP treatment, we attempted to block this responsive signaling pathway by combining ICIs with LP treatment (figure 5A). LP monotherapy and dual combination therapy, either LP and αPD-1 or LP and αCTLA-4, moderately suppressed tumor growth; the triple combination therapy of LP, αPD-1, and αCTLA-4 suppressed MC38 tumor growth to the greatest extent, resulting in complete tumor regression (figure 5B). Notably, the triple combination therapy induced complete tumor eradication in most tumor-bearing mice (80.0%), which was not observed in the other treatment groups (figure 5C). Moreover, tumors treated with LP in combination with αPD-1 and αCTLA-4 showed a remarkable increase in proportion of granzyme B^+^ CD8^+^ T cells compared with that in control tumors. Furthermore, iNOS^+^ M1-like macrophages were noticeably enhanced, while Arg-1^+^ M2-like macrophages were not significantly changed (figure 5D). Mouse body weight was not significantly changed after various LP-based combination therapy compared with control. In addition, splenomegaly was not observed in all mice of the control group and combination therapy group (online supplemental figure 1A).

10.1136/jitc-2022-004799.supp1 Supplementary data

To demonstrate the establishment of immunological memory via LP treatment, mice with complete MC38 tumor regression were rechallenged with MC38 or B16F10 tumor cells. Mice that experienced complete regression after combination immunotherapy were completely immune to MC38 tumor regrowth and maintained a tumor-free status, but were not immune to B16F10 tumor cells (figure 5E). Moreover, tumor-free mice exhibited a significant increase in proportion of CD44^+^CD62L^−^ effector memory T cells in CD4^+^ and CD8^+^ T cell compartments compared with that in control mice (figure 5F).

Taken together, these results suggest that LP treatment combined with ICIs further potentiates the therapeutic efficacy of LP, thereby inducing complete tumor regression and tumor-specific immune memory.

### Triple combination immunotherapy of LP, αPD-1, and αCTLA-4 provided a long-term survival benefit and immune memory in melanoma

**Figure 6:**
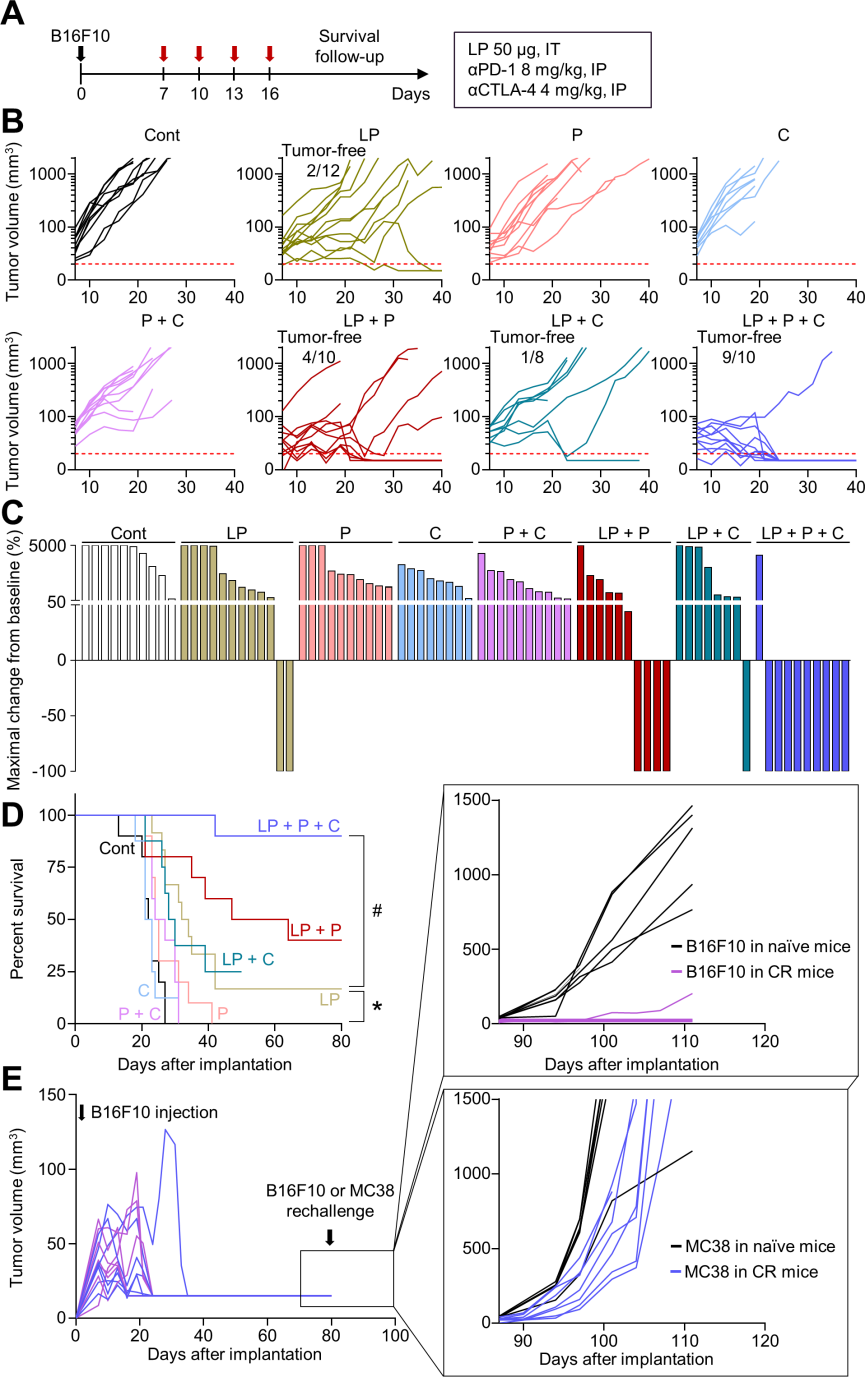
Triple combination immunotherapy improves the survival and enhances the immune memory in melanoma. B16F10 melanoma-bearing mice were treated with LP, αPD-1 (P), and/or αCTLA-4 (C). (A) Schematic diagram of the treatment schedule. (B) Comparison of B16F10 tumor growth. The number of tumor-free mice is indicated. (C) Waterfall plots showing the percentage changes in each tumor volume at the end of the experiment compared with the baseline. (D) Kaplan-Meier curves for overall survival. (E) Comparison of tumor growth after B16F10 or MC38 tumor cells were injected into naïve mice or mice with complete tumor regression. Pooled data from three independent experiments with n=8–12 per group (B–D) and n=6–7 per group (E). *P<0.05 versus control; ^#^p<0.05 versus LP. Log-rank test was performed (D). CTLA-4, cytotoxic T-lymphocytes-associated protein 4; LP, L-pampo; PD-1, programmed cell death protein-1.

To validate whether these findings apply to other tumor models, we evaluated the efficacy of combination therapy in an orthotopic B16F10 melanoma model (figure 6A). Tumor growth was inhibited by 74.3% by LP monotherapy, 56.0% by αPD-1 and αCTLA-4 dual combination therapy, 83.8% by LP and αPD-1 dual combination therapy, 77.9% by LP and αCTLA-4 dual combination therapy, and 95.6% by triple combination therapy compared with that in the controls (figure 6B). Moreover, while LP monotherapy induced complete tumor regression in 16.7% mice, triple combination therapy induced 90.0% complete tumor regression without recurrences (figure 6C). Furthermore, the antitumor effect of the triple combination therapy was associated with a long-term survival benefit: mice treated with triple combination immunotherapy had the highest overall survival rate compared with that of mice in the monotherapy or dual combination therapy groups (figure 6D). In addition, there were no gross toxicities related to combination therapy (online supplemental figure 1B). When mice with complete tumor regression were rechallenged with B16F10 or MC38 tumor cells, they were immune to B16F10 tumors, but not MC38 tumors, indicating tumor-specific immune memory (figure 6E). Therefore, a triple combination could induce long-lasting immunotherapeutic efficacy and prolong the overall survival of mice with melanoma.

## Discussion

LP has been shown to enhance the efficacy of vaccines when used as an adjuvant; however, its efficacy as an antitumor agent remains unknown. Here, we demonstrated that LP, a potent TLR2 and TLR3 agonist, can remodel the tumor immune microenvironment and enhance antitumor immunity. Intratumoral LP-treatment effectively increased tumor-specific activated CD8^+^ T cells within the tumors. In addition, although LP was locally injected, it elicited a potent systemic antitumor immune response in distant tumors. Notably, combination immunotherapy with LP, αPD-1, and αCTLA-4 further enhanced immunotherapeutic efficacy, leading to tumor eradication and long-term survival benefit. Therefore, LP represents an ideal candidate for clinical development in combination cancer immunotherapy.

A previous study revealed the role of LP on CD4^+^ T cell responses as a vaccine adjuvant, especially for HBV vaccine.^24^ However, in our solid tumor models, CD8^+^ T cells and IFN-γ seemed to be more important than CD4^+^ T cells in tumor growth control. Neutralization of CD4^+^ or CD25^+^ T cells did not affect the antitumor efficacy of LP on tumor growth. Therefore, the immunologic mechanisms of LP may differ depending on the type of disease (eg, infection vs cancer).

PRRs are key elements in the activation of the innate immunity and are predominantly expressed on APCs such as macrophages and DCs.^31 32^ Among the PRRs, TLRs show a natural immune-activating capacity; thus, they represent promising targets for improving antitumor immune responses.^33^ TLR stimulation induces the maturation of DCs and activates T cells.^34^ In addition, TLR regulates macrophages polarization, resulting in enhanced antigen uptake by macrophages and T cell activation.^35 36^ For this reason, various TLR agonists, either natural microbial components or synthetic compounds, are under preclinical and clinical development to enhance cancer immunity.^37 38^ Previous studies have reported that TLR4 activation notably increase IFN-γ-secreting CD8^+^ T cells through DC maturation.^39 40^ Furthermore, stimulation of TLR7/8 induces differentiation of myeloid-derived suppressor cells to M1 phenotype macrophages in the TME, thereby suppressing tumor growth.^19 41^

Compared with other TLR agonists, LP has several advantages in inducing an antitumor immune response. First, LP treatment induces a widespread induction of innate immunity by dual activation of myeloid differentiation response-88 (MyD88) and TIR-domain-containing adapter-inducing interferon-β (TRIF).^42^ TLRs transduce immune responses via the adapter proteins MyD88 and TRIF.^43^ All TLRs except TLR3 transmit signaling via MyD88, and TLR3 transmits signaling via TRIF.^44 45^ Since LP simultaneously activates TLR2 and TLR3, it can stimulate both MyD88 and TRIF, thereby inducing extensive activation of innate immunity.^46^ Additionally, activation of TLR3 signaling by LP could trigger robust type I IFN responses, thereby converting poorly immunogenic tumors into T-cell-inflamed tumors, which favorably responded to immune checkpoint blockade.^47^ Further, activation of TLR2 signaling by LP could activate T-cells indirectly and directly. Classically, TLR2 in myeloid cells is known to indirectly enhance T-cell immunity via the activation of antigen-presenting machinery.^48^ Alternatively, TLR2 is also expressed in T-cells and serves as a costimulatory receptor for PAMPs.^49^ Therefore, TLR2 activation by LP may play a critical role in T-cell immunity by directly augmenting antigen-specific Th1 responses.

In the present study, LP was administered intratumorally and was well-tolerated without significant local and systemic toxicities. Moreover, local LP treatment induced strong T-cell-mediated immunity not only in injected tumors, but also in distant non-injected tumors. Therefore, LP might be effective in treating advanced cancers with multiple distant metastases, overcoming the limitations of previously approved TLR agonists, BCG and imiquimod, which are effective only in patients with early-stage localized tumors.^31 50^ Furthermore, triple combination immunotherapy of LP with ICIs showed the highest efficacy, inducing complete tumor regression and prolonging survival.

In this study, we were not able to distinguish the tumor-infiltrating cytotoxic T cells and tumor-resident cytotoxic T cells in the TME. Therefore, further study will be needed to show whether LP directly augments the infiltration of cytotoxic T cells into the TME or LP expands tumor-resident cytotoxic T cells.

In conclusion, our results demonstrated that intratumoral LP treatment effectively enhances both innate and adaptive antitumor immunity within the tumor, thereby potentiating the antitumor efficacy of ICIs. These findings require further validation in future clinical trials.

## Data Availability

Data are available upon reasonable request.
