# Supplementary material for: Intratumoral immunotherapy using a TLR2/3 agonist, L-pampo, induces robust antitumor immune responses and enhances immune checkpoint blockade
Source: J Immunother Cancer. 2022 Jun 28;10(6):e004799. doi: 10.1136/jitc-2022-004799 (PMC9240943; doi:10.1136/jitc-2022-004799)
Supplement: Supplementary data [file jitc-2022-004799supp001.pdf]

Supplemental data

Intratumoral immunotherapy using a TLR2/3 agonist, L-pampo, induces robust anti-tumor immune responses and enhances immune checkpoint blockade

Won Suk Lee, Dong Sung Kim, Jeong Hun Kim, Yoonki Heo, Hannah Yang, Eun-Jin Go, Jin Hyoung Kim, Seung Joon Lee, Byung Cheol Ahn, Jung Sun Yum, Hong Jae Chon, and Chan Kim

Supplemental Figures

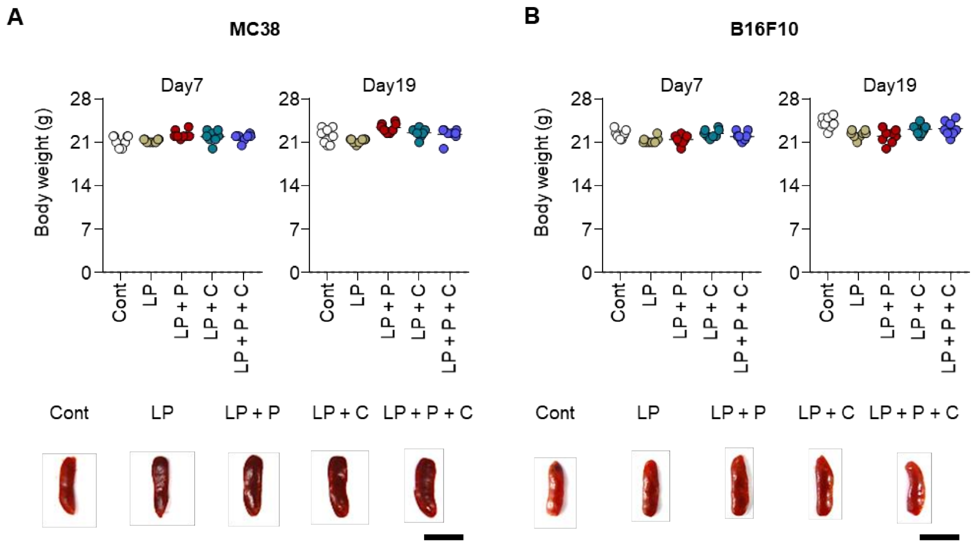

Supplemental Figure 1. LP-based therapy did not show gross toxicities.

In both MC38 colon cancer (A) and B16F10 melanoma (B) models, body weight was not changed after LP-based therapy compared with control and splenomegaly was not observed in all mice. Scale bars, 10 mm.
